# *Symbiodinium*—Invertebrate Symbioses and the Role of Metabolomics

**DOI:** 10.3390/md8102546

**Published:** 2010-09-30

**Authors:** Benjamin R. Gordon, William Leggat

**Affiliations:** 1 AIMS@JCU, Australian Institute of Marine Science, School of Pharmacy and Molecular Sciences, James Cook University, Townsville, Queensland 4811, Australia; 2 ARC Centre of Excellence for Coral Reef Studies, James Cook University, Townsville, Queensland 4811, Australia; E-Mail: bill.leggat@jcu.edu.au; 3 School of Pharmacy and Molecular Sciences, James Cook University, Townsville, Queensland 4811, Australia

**Keywords:** metabolomics, zooxanthellae, marine, Symbiodinium, coral

## Abstract

Symbioses play an important role within the marine environment. Among the most well known of these symbioses is that between coral and the photosynthetic dinoflagellate, *Symbiodinium* spp. Understanding the metabolic relationships between the host and the symbiont is of the utmost importance in order to gain insight into how this symbiosis may be disrupted due to environmental stressors. Here we summarize the metabolites related to nutritional roles, diel cycles and the common metabolites associated with the invertebrate-*Symbiodinium* relationship. We also review the more obscure metabolites and toxins that have been identified through natural products and biomarker research. Finally, we discuss the key role that metabolomics and functional genomics will play in understanding these important symbioses.

## 1. Introduction

Metabolomics is the newest of the ‘omics’ based sciences and is fast becoming a popular tool in many fields of research. The first metabolomics analyses were performed at the turn of the century and, in the decade since, the literature available has gone from a handful published in 2000 [1–3] to more than 600 published in 2009. Metabolomics was first defined by Oliver *et al.* [3] in 1998 as “the quantitative complement of all of the low molecular weight molecules present in cells in a particular physiological or developmental state” [4]. In the years before the advent of metabolomics, the majority of metabolite studies were more focused on the study of specific metabolites or groups of metabolites and how they changed according to a given stimuli or from the effects of an alteration (e.g., a genetic mutation). These types of studies were often classified as metabolite target analysis, metabolic profiling or metabolic fingerprinting [5]. Although definitions can be vague and open to interpretation, the common factor in all three definitions is simply, that none of the methods attempt to study the global suite of metabolites in an unbiased fashion. However, metabolomics has evolved over the last ten years and thus, for the purpose of clarity, it is now more commonly defined, and widely accepted, as the study of endogenous, low molecular weight (<1500 Da), global metabolite profiles in a system (cell, tissue, or biofluid) under a given set of conditions [4,6,7].

Metabolomics is fast becoming a more widely used approach for studying the interaction of living organisms with their environment. Part of its appeal and value lie in the fact that it is complementary with the other “omics” methods such as genomics, transcriptomics and proteomics [8]. The fact that metabolomics is able to report on the actual functional status of an organism which can then be related to its phenotype is a part of the reason that metabolomics has become a popular tool in the environmental sciences. Another factor contributing to its popularity is that metabolomics has the ability to raise questions about the study organism and thereby uncover unexpected metabolite responses and relationships, which can lead to hypothesis generation and further investigation [9,10]. The study of how organisms interact with their environment is a broad field, and not only does this include the more obvious field of ecology but also less obvious fields such as agriculture [11], viticulture [12] and forensics [13].

Cienkowski first described unicellular, photosynthetic dinoflagellates of the genus *Symbiodinium* in 1871 [14], which are more commonly referred to as zooxanthellae (from here on they will only be referred to zooxanthellae in cases where the dinoflagellates have not been clearly identified as *Symbiodinium* spp.). They live freely in the water column and are in symbiosis with a number of marine invertebrates, such as coral, giant clams and anemones, predominantly found in clear tropical waters with low nutrient concentration and plankton densities [15,16]. In this low nutrient environment, some invertebrates have formed this symbiotic relationship with *Symbiodinium* in order to gain a competitive advantage through increased fitness [17], allowing the bilateral exchange of metabolites, including the production of metabolites that are not formed by either organism separately [18]. Of particular interest to researchers over the past few decades, is the relationship of *Symbiodinium* with hermatypic corals. The importance of this relationship cannot be understated due to the fundamental role that hermatypic corals have played in the formation and maintenance of coral reef habitats. These habitats have provided a livelihood for local communities with tourism and fishing industries relying heavily upon them. Unfortunately, due to anthropogenic pollution and global climate change this ecosystem is under increasing threat [19,20].

It is not the intentions of the authors to review the role of metabolomics in the environmental sciences (for a comprehensive review on the topic see Bundy *et al.* [9]). Rather, the purpose of this review is to analyze the past, present and future roles that metabolomics and targeted metabolite analysis have in marine science with a specific focus on *Symbiodinium*-invertebrate symbioses. Rather distinctly, metabolomics has—to the best of the authors’ knowledge—never been utilized for the study of *Symbiodinium*. As such, an in-depth historical analysis of targeted metabolite research of *Symbiodinium* and invertebrate hosts will set the tone for a detailed discussion on metabolomics and systems biology. Furthermore, it is obvious from the literature available that the major metabolic themes in algal-invertebrate symbioses have been involved with the recycling of inorganic carbon compounds between the host and the symbiont and the ability of the symbiont to supplement the hosts’ nutritional demands. It is the intentions of the authors to examine metabolite research of algal-invertebrates symbioses and provide a brief history of where the study of *Symbiodinium* metabolites began (see [20] for a comprehensive review of zooxanthellae metabolites).

## 2. *Symbiodinium* Symbiosis

Symbiosis was originally defined by Anton deBary as “the living together of differently named organisms” [20]. *Symbiodinium* have established intracellular symbioses with corals, anemones, jellyfish, nudibranchs, Ciliophora, Foraminifera, zoanthids and sponges and have extracellular symbioses with giant clams (for review see [21]). The majority of the estimated 798 coral species have established symbiosis with *Symbiodinium*; in this symbiotic relationship the algae are found within a host-derived vacuole (symbiosome membrane) within the gastrodermal cell layer. The cell membrane is derived during the acquisition and division of the algal symbionts, and is analogous to the symbiosome in legumes where the plant membrane encloses the symbiotic rhizobium cells [20]. Roth *et al.* defined this cell membrane in 1988 as the host derived symbiosome [22]. It was not until 1993 that further work by Rands *et al.* [23] analyzed this cell membrane more closely; they found that the cell membrane of the anemone host *Anemonia viridis* controls the availability of phosphorous compounds and other nutrients to zooxanthellae. Moreover, the transportation of all nutrients must proceed via this cell membrane and thus it is critical to the metabolic interaction between the symbiont and host.

*Symbiodinium* cell numbers can range from one symbiont per host cell [24] to over 60 in some hydroids [25], with *Symbiodinium* normal cell densities being in the order of 1–2 million cells cm^−2^ of coral tissue. Although only four *Symbiodinium* species have been formally characterized; they represent a large species complex and, based upon genetic markers [26], at least 160 distinct *Symbiodinium* strains have been identified, each of which may represent a distinct species with distinct physiologies. While some *Symbiodinium* strains are considered generalists, and can establish symbiosis with more than one species of host, others are restricted in the symbiotic association they can form. Given the diversity in both coral host species and algal genotypes there is a large potential for a range of metabolic relationships.

## 3. Targeted Metabolite Analysis

### 3.1. Nutritional Roles of *Symbiodinium*

Much of the metabolite analysis of *Symbiodinium* began in the mid 1950s after Zahl and McLaughlin reported a method for isolating and cultivating these dinoflagellates [27]. It was from that point onwards that studies of *Symbiodinium* metabolites accelerated. In 1958, Muscatine and Hand [28] were the first to suggest, based on experimental evidence, that *Symbiodinium* provide nutrition to their anemone host. In that study, sea anemones with symbiotic dinoflagellates were exposed to seawater containing ^14^C labeled CO_2_ for 18 and 48 hours. After fixation of the labeled carbon, autoradiograph of dissected sections of the host showed conclusively that labeled metabolites moved from the algae to the tissue of the anemone host after 48 hours of exposure but not after 18 hours. Muscatine *et al.* [29] then demonstrated that symbiotic *Symbiodinium* produce relatively large amounts of soluble carbohydrates in contrast to free living *Symbiodinium*, which produced small amounts of glycolic acid.

**Figure 1:**
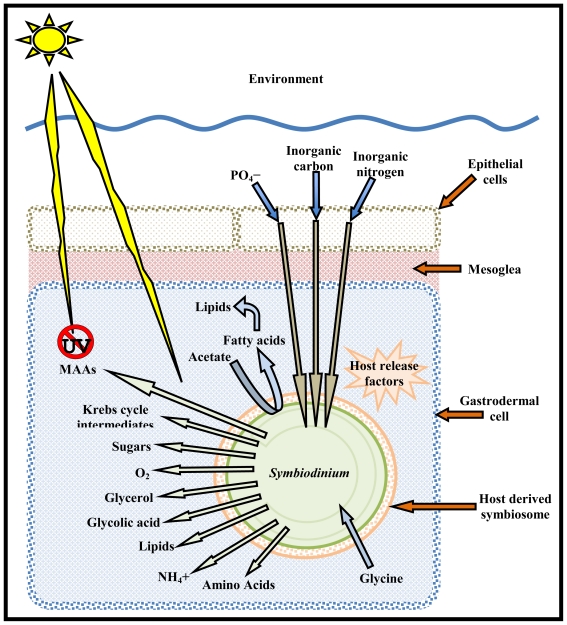
Schematic diagram of the invertebrate-*Symbiodinium* relationship.

By the 1970s, researchers had firmly established that *Symbiodinium* release soluble products of photosynthesis, which were utilized by their hosts, along with identifying a variety of those metabolic products from sugars and amino acids to esters, alcohols and lipids (see Table 1 and Figure 1) [30–32]. Most research during this period emphasized the nutritional interactions between the host and symbiont. Of particular interest was the role of *Symbiodinium* in translocating photosynthetic products and the recycling of host metabolic products, such as ammonium and phosphate. Once again, the majority of research made use of labeled carbon to identify the metabolic products produced and recycled by *Symbiodinium*.

Although there was direct evidence that photosynthetic products were chemically incorporated into the tissue of the host, little was known in the early 1970s about the quantity of metabolites produced by the symbiont, nor how the host utilized them. It was in this context that Trench published three papers on his research in 1971 [49–51]. The first paper examined the movement of labeled carbon in the sea anemone, *Anthopleura elegantissima* and the zoanthid *Palythoa townsleyi*. Chemical extraction and paper chromatography methods were used to fractionate the low molecular weight water-soluble compounds from the lipids, proteins and nucleic acids. In essence, the photosynthetically fixed carbon (predominant as glycerol) was incorporated into the animal tissue primarily as lipids and proteins [49]. In the second paper of the series [50], Trench looked more closely at the type of compounds that were produced by *Symbiodinium*. Using similar chemical and chromatographic methods as in his first paper, he identified a number of different compounds. In particular, it was shown that glycerol was the major extracellular product and other labeled compounds included alanine, glucose, fumaric acid, succinic acid, glycolic acid and two other unidentified organic acids. One of the more noteworthy observations made by Trench in this paper was that although different *Symbiodinium* isolates produced similar compounds, they were in significantly differing proportions. Trench concluded that although *Symbiodinium* from different hosts were morphologically the same, they did differ biochemically. This was probably the first observation made which alluded to the now well known fact that *Symbiodinium* spp. are a diverse group of dinoflagellates. Moreover, his observation was consistent with the facts we have today about how dynamic the metabolome can be. The third and final paper in the series looked at the effect of host tissues on the excretion of photosynthetic products *in vitro* by *Symbiodinium*. It was observed that *Symbiodinium* excrete a greater amount of metabolites when incubated in a homogenate of host tissue than *Symbiodinium* that were incubated in seawater. Furthermore, Trench found that when incubated in a homogenate of host tissue from algal free animals, there was no observable increase in the production of extracellular metabolites. However, when algal free animals were infected with *Symbiodinium*, the host tissue homogenate then increased the production of metabolites. These results suggested that the host plays an important role in regulating the type, and quantity, of metabolites produced by *Symbiodinium*. This series of papers by Trench clearly highlights the fact that *Symbiodinium* differ metabolically and hence, so too would their biosynthetic pathways.

The concept of the host controlling the release of metabolites from the symbiont has been formalized in the concept of “host factors” (HF) or “host release factors” (HRF)[34,51], although there are still some questions as to their exact identity. Gates *et al.* [52] proposed that host free amino acids (FAA), themselves metabolites, served as HRFs. They found that a variety of FAAs stimulated *Symbiodinium* carbon fixation up to two fold and carbon release up to four fold. Gates *et al*. tentatively identified the released compounds as glucose, glycerol, alanine, glycine, serine, glutamine, valine, phenylalanine, leucine, citrate, glycerate, glycolate, lactate and succinate. In contrast to this study, a number of other papers have been unable to reproduce similar results [53,54], and instead have proposed that other small (<1000 Da) unknown host compounds must act as HRFs [55]. In addition to compounds that stimulate the release of metabolites, it has been proposed that corals may also produce a low molecular weight (<1000 Da) peptide that can inhibit *Symbiodinium* photosynthesis [56].

Although coral tissue can consist of up to one-third lipid by dry weight, it was not until 1977 that Patton *et al.* [38] provided evidence that *Symbiodinium* primarily performed the role of lipid synthesis in corals from host derived acetate. They also postulated that energy transfer from symbiont to host via acetate recycling might be the key to the ecological success of corals in nutrient poor waters. As such, they proposed that the digestive and degradative metabolism of the host produced the acetate from exogenous food sources, which was then transferred and subsequently absorbed by the *Symbiodinium*. At which point the *Symbiodinium* would reduce the acetate molecules to fatty acids in the chloroplast using excess adenosine triphosphate. The triglyceride fatty acids were transferred back to the host via “lipid bodies” that were formed on the outer surface of the *Symbiodinium* cell wall. It was proposed by Patton *et al.* that the surface membrane of the globules was the site of wax ester and triglyceride synthesis. Recent research has shown that these lipid bodies exist within both the host and symbiont cells and this is discussed in more detail below. Patton *et al.* found that wax esters and triglycerides comprised 75% of the intact coral lipids whereas the symbiont comprised of only about 8% of these neutral lipids. Conversely, structural lipids such as sterols, phospholipids and galactolipids made up approximately 67% of the symbiont lipids but only 16% of the host lipids. Furthermore, they found that labeled fatty acids derived from acetate were more unsaturated than endogenous fatty acids, which implied that the host either saturated the fatty acids or the transfer process was selective for saturated fatty acids. The results showed that the majority of the coral’s reserved energy (wax esters and triglycerides) was located within its own tissue and that *Symbiodinium* were the primary producers of these lipids that were manufactured from host derived acetate.

More recently, research on lipids has delved deeper into the function and formation of lipid bodies in mammalian cells. In fact, a review by Martin and Parton in 2006 [57] described these lipid bodies as pivotal cellular organelles with specific structural and functional characteristics. They consist of a core of neutral lipids predominantly comprised of triacylglycerols or cholesteryl esters, which are surrounded by a monolayer of phospholipids and associated proteins. Functionally, these mammalian lipid bodies are now regarded as complex organelles involved in a number of cellular processes such as, cell signaling [58], and visual chromophore regeneration [59] and as previously discussed, the lipid metabolism in *Symbiodinium*. Lipid bodies involved in the *Symbiodinium*-coral symbiosis were analyzed more closely in 2009 by Luo *et al.* [60]. They attempted to test the hypothesis that lipid bodies in *Symbiodinium* and the host gastrodermal cells of *Euphyllia glabrescens* were unique organelles that reflect the dynamic nature of the endsymbiotic status. Using dual-emission ratiometric imaging with a solvatochromic fluorescent probe, they were able to show that the ratio of polar *versus* neutral lipids in lipid bodies of the host, increased upon bleaching. Thus, indicating a decrease in neutral lipid accumulation within the gastrodermal cell. Conversely, neutral lipid accumulation increased in the lipid bodies of the symbiont when bleached. The work performed by Luo *et al.* clearly demonstrated that the composition and morphology of lipid bodies was positively correlated to the endosymbiotic status and hence, implicated the lipid bodies in lipid trafficking between the host and the symbiont for the purpose of regulating the endosymbiosis. Lipids are not only essential for energy storage and nutrition, but they also have an important role to play as structural components of symbiont cells. As such, differences in lipid composition can have distinct effects on the ability of different *Symbiodinium* species to adapt to changes in their environment. In 2004, Tchernov *et al.* [61] demonstrated that thermal stress sensitivity of *Symbiodinium* could be categorized by the level of lipid saturation and lipid stacking patterns in the thylakoid membrane. Gas Chromatography Mass Spectrometry (GCMS) analysis of several *Symbiodinium* isolates revealed a striking contrast between the thermally tolerant and sensitive isolates. The thermally tolerant *Symbiodinium* had much lower levels of the major polyunsaturated fatty acid, Δ6,9,12,15-*cis*-octadecatetraenoic acid (18:4), in relation to Δ9-*cis*-octadecatetraenoic (18:1). Furthermore, transmission electron micrographs of thermally sensitive *Symbiodinium* exposed to higher temperatures, showed a significant disruption in the organized stacking pattern of the thylakoid membrane, which is essential for efficient photochemical energy transduction. Based on their evidence, Tchernov *et al.* were clearly able to demonstrate that thylakoid membrane lipid composition is a key determinate of thermal stress sensitivity in the symbiotic algae of cnidarians.

Given the nutrient limitation seen in tropical waters, the translocation of organic nitrogen, in the form of amino acids, from *Symbiodinium* to the coral is extremely important. *Symbiodinium* are able to recycle waste nitrogen produced by the host to synthesize a number of essential amino acids that the host is unable to. Until 1999 substantial evidence for the transport of essential amino acids from *Symbiodinium* to the host had yet to be observed. Wang and Douglas [62] reasoned that previous experimental designs that explored labeled photosynthate release, would fail to detect amino acid transfer to the host tissue if the amino acids were released many hours, or even days, after carbon fixation took place; or that amino acids were not synthesized by photosynthetically derived carbon. Hence they examined, over several days, the metabolic fate of labeled carbon compounds given to the symbiosis. Using extended pulse chase experiments in the order of two days, they were able to provide direct evidence for the transport of a number of essential amino acids from *Symbiodinium* to the host.

Both the coral and algae are capable of assimilating ammonium from their environment in addition to producing it metabolically via the enzymes, glutamate dehydrogenase and glutamine synthetase. This ability of the *Symbiodinium*-invertebrate relationship is thought to be a major reason for their success in nutrient poor environments. Muscatine and D’Elia studied the uptake, retention and release of ammonium in reef corals in order to provide “evidence to support the hypothesis that combined nitrogen is recycled within the coral-algae symbiosis” [63]. Their experiments provided clear results that symbiotic corals uptake and retain ammonium, and that the process was enhanced by light. In contrast to the symbiotic corals, non-symbiotic corals released ammonium into the medium under all conditions. In view of their results, they tentatively concluded that the uptake and retention of ammonium was due to the activity of *Symbiodinium*. This process was considered as an adaptation to a deficiency of environmental nitrogen since virtually all of the ammonium excreted by the coral host was taken up by the symbiont. D’Elia *et al.* [64] further supported the findings by Muscatine in 1983 where they proposed a depletion-diffusion mechanism for the uptake of ammonium, whereby the symbiont was responsible for the majority of ammonium assimilation. However, ammonium assimilation is not that simple and contrasting studies have supported the theory that the host may be limiting the supply of ammonium as a method of controlling the population of *Symbiodinium*. For example, Wang and Douglas [65] conducted a set of experiments that studied the assimilation of ammonium by the host under dark conditions. They found that ammonium assimilation along with protein and free amino acid pool sizes were the same in dark conditions, compared with illuminated conditions, after supplementing the medium with organic carbon compounds. Their evidence strongly suggested that the host controls the concentration of ammonium, via enhanced ammonium assimilation and, furthermore, contradicts the findings by previous researches that the symbiont was primarily responsible for ammonium assimilation.

Unlike the invertebrate host, *Symbiodinium* is capable of utilizing nitrate as a nitrogen source. The first evidence of this was when reef corals were found to have a light independent mechanism for the uptake and reduction of dissolved nitrate from seawater [66] and that nitrite was present in both the coral and symbiont tissue. Further evidence for the uptake of nitrates by *Symbiodinium* has been shown by the discovery of expressed sequence tags for transporter enzymes of both nitrate and nitrite [67], along with findings that show an increase in *Symbiodinium* cell densities upon increases in nitrate concentration and uptake [68]. Although there is evidence for assimilation of nitrogen by both the host and symbiont, this interplay is yet to be clearly elucidated.

### 3.2. Diel Cycles

Given the importance of photosynthesis to the coral symbiosis, it is not surprising that there are a number of molecules, proteins and metabolites that exhibit large diel variations. One of the major diel changes seen in symbiotic invertebrates is intracellular oxygen tension, which is derived from *Symbiodinium* photosynthesis. Using oxygen microelectrodes, Dykens and Shick [43] measured the partial pressure of pure oxygen bubbles on the surface of symbiotic sea anemones and found that the anemones were subjected to a continuous flux of hyperbaric oxygen. Similar studies have estimated that O_2_ concentrations at the tissue surface vary from less than 2% of air saturation during dark periods to over 250% saturation in the light. Concomitantly, tissue pH varies from 8.5 in the light to 7.3 in the dark [69]. These high O_2_ concentrations have profound implications for the coral host given molecular oxygen undergoes reductions to form oxygen radicals, which are particularly destructive to cells. One of the important enzymes involved in reactive oxygen detoxification is superoxide dismutase (SOD), this enzyme catalyzes the dismutation of two oxygen radicals to produce hydrogen peroxide and molecular oxygen. Dykens and Shick found that anemones with higher chlorophyll content had higher rates of SOD activity, indicating that the host was altering its protein expression in response to the *Symbiodinium* physiology/metabolism. In addition, exposing anemones to exogenous hyperbaric oxygen caused a 62% increase in SOD activity while anemones kept under dark, photosynthetically poor conditions had reduced levels of SOD activity. Subsequently Levy *et al.* [70] examined the wavelength dependence of two free radical scavenger enzymes, SOD and catalase (CAT). They found that in the animal host the enzyme response to the spectral distribution of light was higher than that of the zooxanthellae, probably due to accumulation of free radicals within the host tissue. Furthermore, they found that the activity of the enzymes was affected by the length of the day and night cycles, and in the laboratory, by the duration of the illumination. The activity of these scavenger enzymes such as SOD, are vital in acclimatization and survival of corals in shallow water environments with high light radiation as they reduce the effects of oxidative damage to cells by free radicals.

While there have been no direct measurements of diel changes in metabolite transfer in corals, there is clear evidence in other symbioses that, unsurprisingly, the amount of photosynthetically fixed carbon moved to the host, varies over the day. In the giant clams symbiosis, where sampling the hemolymph (blood) can be easily performed, there is ample evidence that glucose is the major carbohydrate transported to the host with concentrations varying from less than 100 μM in the dark to almost 400 μM at noon [71,72].

**Scheme 1:**
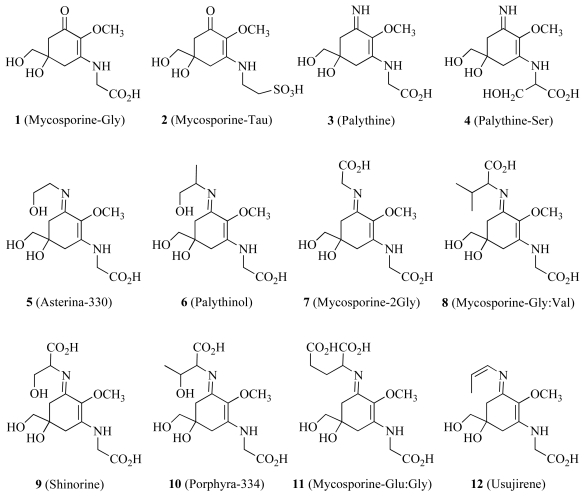
Molecular structures of some common mycosporine-like amino acids in marine organisms.

In addition to driving photosynthesis, high light levels expose the host and symbiont to damaging UV radiation. One particular defense against UV is the production of mycosporine-like amino acids (MAAs). These compounds (**1**–**12** in Scheme 1) are characterized by a cyclohexanone or cyclohexenimine chromophore conjugated with a nitrogen substituent of an amino acid and absorb UV radiation without any further photochemical reactions [73]. The source (host *vs.* symbiont) of these compounds is not clear and concentrations in coral tissue can vary according to fluctuations in light levels. For example, research performed by Yakovleva and Hidaka [74] in 2004 was able to show that freshly isolated *Symbiodinum* contained no MAAs yet they were distributed throughout the animal tissue. In contrast, work by Banaszak *et al.* [75] in 2000 showed that cultured *Symbiodinium* cells of clade A did produce MAAs, but not cells from from clades B and C. Increases of up to two fold at midday have been demonstrated for discrete MAA species, in particular the concentration of imino-MAA species varied in response to light while mycosporine-glycine (**1**) did not [74]. Some MAAs also have the potential to act as free radical scavengers [46]. For example, the research by Dunlap and Yamamoto [46] of small-molecule marine antioxidants elucidated six common MAAs from four different marine species. The MAAs isolated included five cyclohexenimine MAAs (shinorine **9**, porphyra-334 **10**, palythine **3**, asterina-330 **5** and palythinol **6**) and a single cyclohexanone MAA (mycosporine-glycine **1**). Upon studying the oxidation properties of each of these MAAs they found that the oxidative robustness of imino-MAAs was in keeping with their sunscreen properties and thus had no definitive antioxidant activity, whereas mycosporine-glycine did have moderate antioxidant activity.

The production of MAAs has been shown to correlate directly with the availability of ammonium in the red algae *Porphyra leucosticta* and *Porphyra umbilicalis* [76]. This study evaluated the effect of ammonium availability on photosynthesis on the basis of fluorescence of chlorophyll *a*, photosynthetic pigments and MAA content. Discs of both species were cultured with three ammonium concentrations (0, 100 and 300 μM) under artificial light. Four MAAs were identified in both *Porphyra* species (shinorine **9**, porphyra-334 **10**, palythine **3** and asterina-330 **5**) and in both cases MAA concentration increased at high ammonium concentrations and exposure to UV radiation. Conversely, photosynthetic activity decreased under the culture conditions due to UV radiation and ammonium availability.

### 3.3. Biomarkers and Natural Products

The concept of using chemical fingerprints to classify different phenotypes of corals was first explored in 1982 [77]. That study focused on marine natural products involving secondary metabolites from soft corals and sponges from the Red Sea. These organisms were found to be chemically protected from predation by fish and bacterial infection, and a potential source of bioactive pharmaceuticals. However given the difficulty in species identification of soft corals, Kashman *et al.* investigated whether chemical fingerprints of sesquiterpenes and other volatiles obtained through gas chromatography (GC) and liquid chromatography (LC) could be used as a complementary tool for phenotype identification. Considering that chemotaxonomy was a novel concept at the time, Kashman *et al.* did notice unique chemical differences upon visual analysis of the chromatograms of six *Sinularia* species and were actively using them to assist traditional taxonomists in defining different species, especially where doubt existed due to different growth forms.

The use of biomarkers has also been examined in *Symbiodinium* symbioses by Cuif *et al*. [78] who isolated compounds greater than 3 kDa from coral skeletons of symbiotic and non-symbiotic corals, and then performed derivitizations of proteins and hydrolysis of polysaccharides to isolate amino acids and monosaccharides. Several statistical analyses were used in order to find the most significant amino acids and monosaccharides that were able to distinguish between soluble matrices of symbiotic and non-symbiotic corals. The analyses included univariate statistical methods to elucidate significant variables and these included a one-way ANOVA, a parametric t-test (α = 5%) and a non-parametric Kolmogorov-Smirnov test (α = 5%). Multivariate principal component analysis (PCA) was then used to represent the statistical weights of all the significant variables and the distribution of the individuals according to their overall biochemical contents. The amino acids that were identified by the univariate tests included glutamic acid, alanine, serine, threonine and histidine, and the monosaccharides included galactose, mannose, galactosamine, glucosamine and arabinose. Once the discriminant amino acids and sugars were identified (a total of nine variables), they were combined into a single data set for the multivariate PCA. The first three principal components (PCs) of the PCA explained 80% of the total variance of the data set where PC 1 explained 50.1%, PC 2 explained 18.7% and PC 3 explained 11.1%. Of the three components, PC 1 was best explained by galactosamine and serine, thus suggesting that the two compounds gave the best explanation (50.1%) of differences in symbiotic and non-symbiotic corals. In essence they found that symbiotic corals had high mannose, galactose, arabinose, glutamic acid and threonine contents whereas non-symbiotic corals had high alanine, serine, galactosamine and glucosamine contents. These results demonstrated that the metabolites of symbiotic and non-symbiotic corals were different and could be recognized through the particular ratio of certain amino acids or sugars in soluble skeletal matrices.

**Scheme 2:**
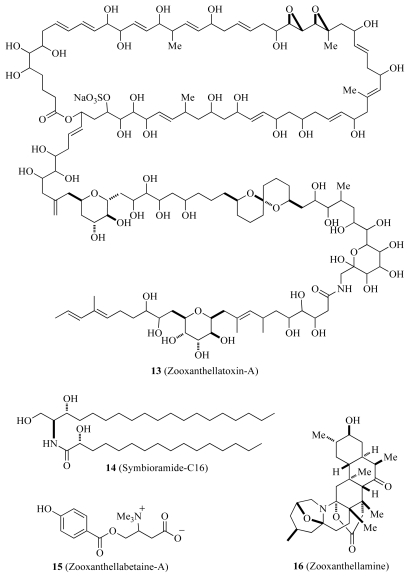
Molecular structures of zooxanthellatoxin-A (**13**), symbioramide-C16 (**14**), zooxanthellabetaine (**15**) and zooxanthellamine (**16**).

One of the first studies to investigate novel natural products from *Symbiodinium* was performed in 1993 by Nakamura *et al.* [47]. Upon isolating zooxanthellae from the flatworm *Amphiscolops* sp., they reported the first examples of water-soluble large molecules that induced prominent contraction of rabbit aorta. The active compounds were identified and named as zooxanthellatoxins (ZTs) A (**13**) and the congener ZT-B (see Scheme 2). Initial characterization of these toxins clearly showed that they were unique in that they have more double bonds and fewer ethereal rings compared to similar toxins isolated from different species of dinoflagellates. Structural similarities between ZTs and palytoxins suggested that there exist common biogenetic processes, such as the polyketide pathway, utilizing a glycine starting unit and tetrahydropyran ring formation. Their results were suggestive of the potential usefulness of *Symbiodinium* as a source of bioactive material, and additionally, further suggestive that they may in fact be the source of particular toxins isolated from symbiotic marine invertebrates.

Further natural products work on *Symbiodinium* by Nakamura *et al.* [48] elucidated four new compounds from ethanol extracts of cells cultured under varied conditions. Two of these four were betaines, zooxanthellabetaine-A and -B (**15**) another was the C30 alkaloid, zooxanthellamine (**16**), and the last was a new ceramide, symbioramide-C16 (**14**) (see Scheme 2). It was found that zooxanthellamine had a very similar alkaloid structure to a compound obtained from zoanthids (zoanthamine), which suggested this compound was algal, and not host derived [48]. Their work highlighted an interesting detail about the production of metabolites in *Symbiodinium*, namely, that metabolites and the bioactive molecules that they produce could theoretically be manipulated by culture conditions and host factors to produce a variety of different metabolites of interest to natural products researchers.

**Scheme 3:**
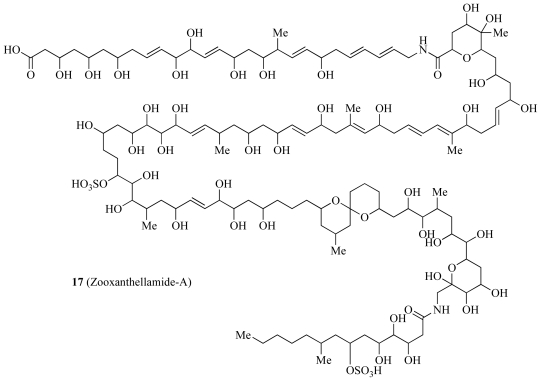
Molecular structure of zooxanthellamide-A (**17**).

A number of compounds related to ZTs have also been isolated from *Symbiodinium.* Zooxanthellamide A (**17**, Scheme 3) was purified from free-living zooxanthellae found in a Hawaiian tidal pool [44]. Zooxanthellamide A has a smaller molecular weight than that of ZTs and the structure differs considerably. Unlike ZTs, zooxanthellamide A does not possess bisepoxide and exomethylene. There is a pair of both the amide and sulfate groups in zooxanthellamide A, whereas these only exist as lone groups in ZTs. These similarities suggest that ZTs and zooxanthellamide A arise from similar biosynthetic pathways. Subsequently a δ-lactone derivative of zooxanthellamide A was found, zooxanthellamide B [45]. These two compounds led Onodera *et al.* to propose that zooxanthellamides were a novel family of large polyhydroxy metabolites of zooxanthellae due in fact to their significant structural differences compared to ZTs. This showed that the polyhydroxy metabolism of zooxanthellae is in fact quite diverse, however, the biosynthetic pathway has yet to be identified.

## 4. Marine Metabolomics

The application of metabolomics to corals and their symbionts is somewhat limited. In fact, the only paper to date, and to the best of the authors’ knowledge, that applies metabolomics to any species of coral is the research performed in 2009 by Boroujerdi *et al.* [79]. While not exactly looking at the metabolome of corals or *Symbiodinium*, they did look at the metabolome of the temperature dependant coral pathogen *Vibrio coralliilyticus*. The bacterium has been linked to coral disease worldwide, and like many other *Vibrio* species, it exhibits a temperature-dependent pathogenicity. Their work aimed to study the temperature dependence of *V. coralliilyticus* using Nuclear Magnetic Resonance (NMR) spectroscopy and PCA in order to identify metabolites that were either up-regulated or down-regulated due to applied heat stressors. The bacteria were cultured in the lab at two different temperatures (27 °C and 24 °C) and the intracellular metabolites were extracted using a methanol-water technique. The NMR spectra were subjected to PCA, and in doing so they were able to conclude that there were distinct metabolic differences between the virulent (high temperature) and threshold-of-virulency (low temperature) forms of *V. coralliilyticus*. More specifically, they found that betaine, succinate and glutamate were identified as the metabolites that caused the greatest temperature-based separations in the PC scores plots. With increasing temperature, betaine was shown to be down-regulated, while succinate and glutamate were up-regulated. However, during their work they noticed significant inter-batch variability. Upon re-extraction, instrumental and statistical reanalysis, they were able to exclude any systematic errors that may have caused the inter-batch variability and thus, they concluded that there must have been significant biological variability during the growth of *V. coralliilyticus*.

This highlights an important, and sometimes overlooked, natural variation of metabolites within conspecifics, in particular in the metabolomics analysis of ecologically relevant samples. In fact, experimental design and method development is a critical step in all metabolomics work and this has led to the publication of a proposed set of minimum reporting standards for large scale metabolomics work by the Metabolomics Standards Initiative (MSI) [80]. These minimum standards “provide a biological and empirical context for the data, facilitate experimental replication, and enable the re-interrogation and comparison of data by others” [80]. The proposed standards put forth by MSI addresses specific issues in metabolomics research such as, sample preparation, experimental analysis, quality control, metabolite identification, and data pre-processing. An example of the importance of these issues was highlighted by the work performed by Hines *et al.* [81]. Their work made recommendations for field sampling of wild organisms in order to retain a snapshot of the true metabolome at the time of sampling. They proved, quite definitively on their metabolomics study of the marine mussel, *Mytilus galloprovincialis*, that keeping mussels under controlled laboratory conditions produced metabolic perturbations to their normal phenotype that completely masked the response to hypoxic stress. Ergo, the recommendations they made included, dissecting as soon as possible after collection and to determine, as far as possible, the genetic and phenotypic traits of the study organism in order to facilitate the interpretation of data. For example, by determining the gender of marine mussels they were able to facilitate the reduction of metabolic variability amongst their study group.

Given the lack of metabolomics studies on coral-*Symbiodinium* associations it is unsurprising that there is no large-scale datasets on how metabolite profiles vary. The importance of these daily variations in algae metabolites was clearly demonstrated in a toxicity analysis of the unicellular freshwater alga *Scenedesmus vacuolatus*. Kluender *et al.* [82] utilized synchronized algal populations and extracted low molecular weight hydrophilic and lipophilic metabolites from the algae, which were analyzed using gas chromatography mass spectrometry (GCMS) and subsequent multivariate analysis to identify time related patterns associated with growth responses to an applied toxin. The toxin utilized in their research was the photosystem-II inhibiting herbicide prometryn, as it was a model compound, which had a recognized mode of action. As a result, they were able to identify clear time-related trends in metabolite levels over a 14-hour growth period along with distinct statistical separations between control algal populations and those exposed to the toxin. Furthermore, they compared their results to observation parameters used in phytotoxicity assessment and were able to show that metabolite levels responded more rapidly to exposure to the toxin than growth, or lack thereof. The result was a successful proof of their concept that metabolomics could be used as a suitable tool in ecotoxicology and the study of unicellular aquatic algae.

Metabolomics may be able to fill the gaps of symbiotic coral research and obvious gaps exist. For example, considerable effort has been placed on studying the interaction of *Symbiodinium* with their coral host when subjected to environmental changes such as elevated sea temperatures. In particular, there has been a body of recent work directed at the shifts in symbiont composition in a coral colony in favor of *Symbiodinium* that are physiologically more suited to the prevailing environmental conditions. It is believed that such shifts in symbiont composition increase the fitness of the holobiont [83]. The main focus of recent work, in relation to shifts in symbiont composition, has been on the relative resistance of the photophysiology of clade D to thermal stress and how this symbiont can survive elevated sea temperatures [84,85]. However, evidence has emerged that these more thermally tolerant symbionts may transfer significantly less metabolites and impose a higher metabolic costs on their host which leads to reduced host growth [85,86], this is an obvious area in which metabolomics can be utilized.

## 5. Metabolomics in Systems Biology

Systems biology is the “global and integrative study of biological systems and is linked to various kinds of high-throughput omics that have made remarkable contributions to understand biological systems and helping understand the cells as systems” [87]. Determining the biological function of metabolites and, by association, its cognate enzyme and enzyme-encoding gene, can be a complex and daunting task. An often-used strategy involves perturbing a system by systematically introducing genetic variations and observing its phenotypic effect [5]. Thus, it is essential to be able to identify the phenotype correctly and a common approach is to study the cellular responses at the transcript or protein levels. Transcriptomics provides the best coverage of genome level responses, and available data is increasing for both the coral host [88,89] and symbiont [67,90], but limitations in analytical precision and relatively high costs continue to hinder the approach. On the other hand, proteomics is well established and comparatively inexpensive but until highly automated systems for proteomics are refined, the approach will continue to be too focused on the most abundant alterations. Unlike transcriptomic and proteomic approaches, metabolite analysis is far more mature and analytical precisions are often less than 1% relative standard deviations.

Reversing the path of traditional genomic analysis from DNA sequencing to identifying biological function is an approach to systems biology called functional genomics. This approach attempts to reveal the role of genes discovered by determining the complete genome sequence of an organism. Genomics, transcriptomics, proteomics and metabolomics are integrated into functional genomics in order to systematically analyze the function of genes and reduce the complexity of such an approach. One particular advantage metabolomics has in the role of functional genomics is the relative simplicity of the metabolome when compared to the genome. For example, the single celled eukaryote *Saccharomyces cerevisiae* has less than 600 low-molecular weight metabolites compared to some 6000 protein-encoding genes [2]. However, there is no direct relationship between metabolites and genes in the way there is for mRNAs and proteins. Another advantage metabolomics provides the functional genomics analyst is the ability to identify silent mutations. These silent mutations may have no overt phenotypic effects however they will often present themselves as a change in the metabolome. This strong but indirect relationship between the metabolome and the genome makes metabolomics approaches amenable for functional genomics.

These issues highlight the functional genomics work performed by Raamsdonk *et al.* [2] where they exploited the genes of known function in yeast in order to elucidate the roles of unstudied genes. Their approach was termed FANCY for functional analysis by co-responses in yeast. They demonstrated that the FANCY technique was able to reveal the role of genes that produced no overt phenotype when deleted from the yeast genome. The basic premise of the approach is that the growth rate of a deletant may not change because the concentrations of intracellular metabolites change in order to compensate for the effect of the mutation. Co-response coefficients were a central and important aspect of their research. In essence, these co-response coefficients analyze the steady state response of two variables (e.g., internal metabolite concentrations) to the change of a system parameter (e.g., a mutation in a silent gene). Raamsdonk *et al.* have explained this rather eloquently; “When a (silent) gene encoding a product of unknown function is deleted, the resulting co-response coefficient profile will be similar to that of a strain that is deleted for a (known) gene acting on the same functional domain of the cell. Should the gene product act in another part of [the] metabolism, the metabolite concentrations will change in a different way, or will not change at all, resulting in a different co-response coefficient profile”. Moving on, they chose to examine the separate deletion of two genes in yeast, namely the PFK26 and PFK27 genes. They showed that these two genes were silent in terms of growth rate phenotype but they did have a phenotypic effect on the metabolome. Furthermore, because the two genes encoded the same enzyme, they had very similar metabolome phenotypes. Thus, they were able to demonstrate that had the function of one gene been unknown, they could have identified its function based on the function of the other. Metabolomics analysis was also critical in their research. The advantage in this case, was that it was not necessary to predetermine which metabolites were affected by the mutations. By taking snapshots of the metabolome using NMR spectrometry and grouping them together using multivariate statistical analyses, genes of unknown function could be grouped together with genes of known function. The work performed by the Raamsdonk group really was ahead of its time considering how young the field of metabolomics and functional genomics were in 2001. Not only were they able to prove that silent genetic mutations could be identified based on an organism’s metabolome but also, they showed definitively that metabolomics and co-response coefficient profiles could be employed in identifying genes of unknown function. Their approach has the potential to be employed in identifying gene function in a variety of different organisms.

Another interesting and more recent piece of work was that performed by Santos *et al.* [91]. They incorporated a combined systems biology and omics approach to examining the toxic effects of copper in a fish model. A combination of phenotypic responses and health effects were analyzed in parallel with metabolomics and transcriptomics, thus providing researchers with the opportunity to distinguish between the alterations associated with adverse health effects compared to alterations associated with acclimation to increased copper concentrations. Stickleback fish were exposed to five concentrations of copper and their livers were analyzed using cDNA microarrays and one-dimensional NMR to produce transcriptomic and metabolomic profiles, respectively. Hepatic transcriptome and metabolome changes, as well as phenotypic end-points indicative of health effects were measured. In addition, data was analyzed to search for the molecular effect pathways of copper across the range of exposure concentrations and to identify the molecular pathways associated with adverse health effects. The results of their work uncovered some interesting findings. Specifically, that copper damaged DNA and changes were observed genetically and metabolically and that these changes occurred even at environmentally relevant levels. They found that genes coding for enzymes involved in the biosynthesis of cholesterol were significantly down-regulated in the fish model and the results align closely with results observed in a mouse model of Wilson’s disease. Their work highlights the power of functional genomics to uncover novel biological targets of chemical toxicity in an organism and additionally, may provide a model for information on potential pollutant effects on coral health.

## 6. Concluding Remarks

It is clear from the literature available that metabolite analysis of *Symbiodinium* symbioses peaked in the 1970s and 1980s with a keen interest in the nutritional roles that symbionts provided their hosts and how they interacted. The pioneers in these early days of metabolite analysis made a significant contribution to the knowledge that marine science benefits from today. Little was understood in those early years about the quantity of metabolites produced by the symbiont, nor how the host utilized them. Yet not only did they prove that the symbiont was providing nutrition to the host but also, that free living zooxanthellae behaved differently to their counterparts involved in symbiosis. While work continues to this day on elucidating the role of individual metabolites, the rise of genetic methods in the 1990s and beyond has seen the focus of research into metabolites dwindle in favor of genomic analysis.

However, questions about the feasibility of using metabolic biomarkers to aid in identifying phenotypes were asked as far back as twenty odd years ago when Trench noticed that zooxanthellae were biochemically different. Similar issues still exist today with many questions yet to be answered. For example, problems such as phenotype identification, elucidation of signaling pathways between host and symbiont and correlating gene expression with the metabolome are just a few problems that have yet to be fully addressed in the invertebrate-algal symbiosis. With the acceptance of climate change being a significant threat to coral reefs, it is of the utmost importance that we begin to start answering such questions. These issues have always been around and it is now, with advances in instrumentation, the increasing popularity of metabolomics along with a systems biology approach that these questions may finally be answered.

## Figures and Tables

**Table 1:** Common metabolites of the invertebrate-*Symbiodinium* relationship.

Metabolite	Transport Direction		*Symbiodinium* specific	Function	Reference
	Host to symbiont	Symbiont to host			
Maltose		✓		Nutrition	[33]
Glycerol		✓		Nutrition	[34]
Glucose and other hexoses		✓		Nutrition	[29,35]
Glycolic acid		✓		Nutrition	[29]
Glycine	✓			Nucleotide synthesis and nutrition	[36]
Alanine		✓		Protein formation	[18]
Acetate	✓			Fatty acid synthesis	[37,38]
Fatty acids		✓		Lipid synthesis	[38]
Lipids		✓		Energy exchange	[39]
Lactate		✓		Metabolite	[37]
Succinate		✓		Krebs cycle	[37]
Citrate		✓		Krebs cycle	[37]
Ketoglutarate		✓		Krebs cycle	[37]
Malate		✓		Krebs cycle	[37]
Pyruvate		✓		Glycolysis product	[37]
PO_4_^3−^	✓			Nutrition	[40]
NO_2_^−^, NO_3_^−^, NH_4_^+^	✓			Amino acid synthesis	[40]
Inorganic carbon (*i.e.*, CO_2_, HCO_3_^−^)	✓			Photosynthesis	[41,42]
O_2_		✓		Photosynthesis	[43]
Zooxanthellamide-A and B			✓	Unknown	[44,45]
Mycosporine-like amino acids			✓	UV light and free radical protection	[46]
Zooxanthellatoxins			✓	Unknown	[47]
Symbioramide-C16			✓	Unknown	[48]
Zooxanthellabetaines			✓	Unknown	[48]
Zooxanthellamine			✓	Unknown	[48]

## References

[1] Fiehn O Kopka J Dormann P Altmann T Trethewey RN Willmitzer L Metabolite Profiling for Plant Functional Genomics Nat. Biotechnol 2000 18 1157 1161 11062433 10.1038/81137

[2] Raamsdonk LM Teusink B Broadhurst D Zhang NS Hayes A Walsh MC Berden JA Brindle KM Kell DB Rowland JJ Westerhoff HV van Dam K Oliver SG A Functional Genomics Strategy that Uses Metabolome Data to Reveal the Phenotype of Silent Mutations Nat. Biotechnol 2001 19 45 50 11135551 10.1038/83496

[3] Oliver SG Winson MK Kell DB Baganz F Systematic Functional Analysis of the Yeast Genome Trends Biotechnol 1998 16 373 378 9744112 10.1016/s0167-7799(98)01214-1

[4] Goodacre R Vaidyanathan S Dunn WB Harrigan GG Kell DB Metabolomics by Numbers: Acquiring and Understanding Global Metabolite Data Trends Biotechnol 2004 22 245 252 15109811 10.1016/j.tibtech.2004.03.007

[5] Fiehn O Combining Genomics, Metabolome Analysis, and Biochemical Modelling to Understand Metabolic Networks Comp. Funct. Genomics 2001 2 155 168 18628911 10.1002/cfg.82 PMC2447208

[6] Rochfort S Metabolomics Reviewed: A New “Omics” Platform Technology for Systems Biology and Implications for Natural Products Research J. Nat. Prod 2005 68 1813 1820 16378385 10.1021/np050255w

[7] Viant MR Metabolomics of Aquatic Organisms: The New ‘Omics’ on the Block Mar. Ecol. Prog. Ser 2007 332 301 306

[8] Viant MR Bearden DW Bundy JG Burton IW Collette TW Ekman DR Ezernieks V Karakach TK Lin CY Rochfort S de Ropp JS Teng Q Tjeerdema RS Walter JA Wu H International NMR-Based Environmental Metabolomics Intercomparison Exercise Environ. Sci. Technol 2009 43 219 225 19209610 10.1021/es802198z

[9] Bundy J Davey M Viant M Environmental Metabolomics: A Critical Review and Future Perspectives Metabolomics 2009 5 3 21

[10] Kell DB Oliver SG Here Is the Evidence, Now What Is the Hypothesis? The Complementary Roles of Inductive and Hypothesis-Driven Science in the Post-Genomic Era Bioessays 2004 26 99 105 14696046 10.1002/bies.10385

[11] Dixon RA Gang DR Charlton AJ Fiehn O Kuiper HA Reynolds TL Tjeerdema RS Jeffery EH German JB Ridley WP Seiber JN Applications of Metabolomics in Agriculture J. Agric. Food Chem 2006 54 8984 8994 17117782 10.1021/jf061218t

[12] Rochfort S Ezernieks V Bastian SEP Downey MO Sensory Attributes of Wine Influenced by Variety and Berry Shading Discriminated by NMR Metabolomics Food Chem 2010 121 1296 1304

[13] Ovenden SPB Gordon BR Bagas CK Muir B Rochfort S Bourne DJ A Study of the Metabolome of *Ricinus communis* for Forensic Applications Aust. J. Chem 2010 63 8 21

[14] Cienkowski L On the Production of Spores in the *Radiolaria* Archiv. Fur. Mikroskop. Anatomie 1871 7 396 403

[15] Muscatine L Porter JW Reef Corals: Mutualistic Symbioses Adapted to Nutrient-Poor Environments Bioscience 1977 27 454 460

[16] Smith DC Douglas AE The Biology of Symbiosis Edward Arnold London, UK 1987

[17] Trench RK The Cell Biology of Plant-Animal Symbiosis Annu. Rev. Plant Physiol. Plant Mol. Biol 1979 30 485 531

[18] Lewis DH Smith DC The Autotrophic Nutrition of Symbiotic Marine Coelenterates with Special Reference to Hermatypic Corals. I. Movement of Photosynthetic Products between the Symbionts Proc. R. Soc. Lond. B Biol. Sci 1971 178 111 129

[19] Hughes TP Baird AH Bellwood DR Card M Connolly SR Folke C Grosberg R Hoegh-Guldberg O Jackson JBC Kleypas J Climate Change, Human Impacts, and the Resilience of Coral Reefs Science 2003 301 929 933 12920289 10.1126/science.1085046

[20] Yellowlees D Rees TAV Leggat W Metabolic Interactions between Algal Symbionts and Invertebrate Hosts Plant Cell Environ 2008 31 679 694 18315536 10.1111/j.1365-3040.2008.01802.x

[21] Stat M Carter D Hoegh-Guldberg O The Evolutionary History of *Symbiodinium* and Scleractinian Hosts—Symbiosis, Diversity, and the Effect of Climate Change Perspect. Plant Ecol. Evol. Syst 2006 8 23 43

[22] Roth E Jeon K Stacey G Palacios RD Verma PS Molecular Genetics of Plant Microbe Interactions Homology in Endosymbiotic Systems: The Term “Symbiosome” Proceedings of the 4th International Symposium of Molecular Genetics of Plant—Microbe Interactions Acapulco, Mexico 1988 APS Press Acapulco, Mexico 1988 220 225

[23] Rands ML Loughman BC Douglas AE The Symbiotic Interface in an Alga Invertebrate Symbiosis Proc. R. Soc. Lond. B Biol. Sci 1993 253 161 165

[24] Muscatine L Blackburn A Gates RD Baghdasarian G Allemand D Cell-Specific Density of Symbiotic Dinoflagellates in Tropical Anthozoans Coral Reefs 1998 17 329 337

[25] Fitt WK Cellular Growth of Host and Symbiont in a Cnidarian-Zooxanthellar Symbiosis Biol. Bull 2000 198 110 120 10707819 10.2307/1542809

[26] LaJeunesse TC Investigating the Biodiversity, Ecology, and Phylogeny of Endosymbiotic Dinoflagellates in the Genus *Symbiodinium* Using the ITS Region: In Search of A “Species” Level Marker J. Phycol 2001 37 866 880

[27] Zahl PA McLaughlin JJA Isolation and Cultivation of Zooxanthellae Nature 1957 180 199 200

[28] Muscatine L Hand C Direct Evidence for the Transfer of Materials from Symbiotic Algae to the Tissues of a Coelenterate Proc. Natl. Acad. Sci. USA 1958 44 1259 1263 16590343 10.1073/pnas.44.12.1259 PMC528718

[29] Muscatine L Karakashian SJ Karakashian MW Soluble Extracellular Products of Algae Symbiotic with a Ciliate, a Sponge and a Mutant *Hydra* Comp. Biochem. Physiol 1967 20 1 6

[30] Goreau TF Goreau NI Yonge CM On the Utilization of Photosynthetic Products from Zooxanthellae and of a Dissolved Amino Acid in *Tridacna maxima* f. *Elongata* (Mollusca: Bivalvia) J. Zool 1973 169 417 454

[31] Von Holt C Von Holt M Transfer of Photosynthetic Products from Zooxanthellae to Coelenterate Hosts Comp. Biochem. Physiol 1968 24 73 81 4384601 10.1016/0010-406x(68)90959-6

[32] Cernichiari E Muscatine L Smith DC Maltose Excretion by the Symbiotic Algae of *Hydra Viridis* Proc. R. Soc. Lond. B Biol. Sci 1969 173 557 576

[33] Muscatine L Symbiosis of Hydra and Algae—III. Extracellular Products of the Algae Comp. Biochem. Physiol 1965 16 77 92 4379313 10.1016/0010-406x(65)90165-9

[34] Muscatine L Glycerol Excretion by Symbiotic Algae from Corals and *Tridacna* and Its Control by the Host Science 1967 156 516 519 17730744 10.1126/science.156.3774.516

[35] Bil KY Kolmakov PV Parnik T Titlyanov EA Muskatine L Photosynthetic Products in Zooxanthellae of the Symbiotic Corals *Stylophora pistillata* and *Seriatopora coliendrum* Situated at Various Depths Fiziol. Rast. (Moscow) 1991 38 846 854

[36] Von Holt C Uptake of Glycine and Release of Nucleoside Polyphosphates by Zooxanthellae Comp. Biochem. Physiol 1968 26 1071 1079 4394521 10.1016/0010-406x(68)90027-3

[37] Von Holt C Von Holt M Secretion of Organic Compounds by Zooxanthellae Isolated from Various Types of *Zoanthus* Comp. Biochem. Physiol 1968 24 83 92 4384602 10.1016/0010-406x(68)90960-2

[38] Patton JS Abraham S Benson AA Lipogenesis in the Intact Coral *Pocillopora capitata* and Its Isolated Zooxanthellae: Evidence for a Light-Driven Carbon Cycle between Symbiont and Host Mar. Biol 1977 44 235 247

[39] Kellogg RB Patton JS Lipid Droplets, Medium of Energy Exchange in the Symbiotic Anemone *Condylactis gigantea*: A Model Coral Polyp Mar. Biol 1983 75 137 149

[40] Cates N McLaughlin JJA Nutrient Availability for Zooxanthellae Derived from Physiological Activities of *Condylactis* spp J. Exp. Mar. Biol. Ecol 1979 37 31 41

[41] Muscatine L Lenhoff HM Symbiosis: On the Role of Algae Symbiotic with Hydra Science 1963 142 956 958 17753799 10.1126/science.142.3594.956

[42] Patton JS Battey JF Rigler MW Porter JW Black CC Burris JE A Comparison of the Metabolism of Bicarbonate 14C and Acetate 14C and the Variability of Species Lipid Composition in Reef Corals Mar. Biol 1983 75 121 130

[43] Dykens JA Shick JM Oxygen Production by Endosymbiotic Algae Controls Superoxide Dismutase Activity in Their Animal Host Nature 1982 297 579 580

[44] Onodera K-i Nakamura H Oba Y Ojika M Zooxanthellamide A, a Novel Polyhydroxy Metabolite from a Marine Dinoflagellate of *Symbiodinium* sp Tetrahedron 2003 59 1067 1071

[45] Onodera K-i Nakamura H Oba Y Ojika M Zooxanthellamide B, a Novel Large Polyhydroxy Metabolite from a Marine Dinoflagellate of *Symbiodinium* sp Biosci. Biotechnol. Biochem 2004 68 955 958 15118333 10.1271/bbb.68.955

[46] Dunlap WC Yamamoto Y Small-Molecular Antioxidants in Marine Organisms: Antioxidant Activity of Mycosporine-Glycine Comp. Biochem. Physiol 1995 112 106 114

[47] Nakamura H Asari T Ohizumi Y Kobayashi J Yamasu T Murai A Isolation of Zooxanthellatoxins, Novel Vasoconstrictive Substances from the Zooxanthella *Symbiodinium* sp Toxicon 1993 31 371 376 8503128 10.1016/0041-0101(93)90172-f

[48] Nakamura H Kawase Y Maruyama K Murai A Studies on Polyketide Metabolites of a Symbiotic Dinoflagellate, *Symbiodinium* sp.: A New C30 Marine Alkaloid, Zooxanthellamine, a Plausible Precursor for *Zoanthid* Alkaloids Bull. Chem. Soc. Jpn 1998 71 781 787

[49] Trench RK The Physiology and Biochemistry of Zooxanthellae Symbiotic with Marine Coelenterates. I. The Assimilation of Photosynthetic Products of Zooxanthellae by Two Marine Coelenterates Proc. R. Soc. Lond. B Biol. Sci 1971 177 225 235

[50] Trench RK The Physiology and Biochemistry of Zooxanthellae Symbiotic with Marine Coelenterates. II. Liberation of Fixed 14C by Zooxanthellae *in Vitro* Proc. R. Soc. Lond. B Biol. Sci 1971 177 237 250

[51] Trench RK The Physiology and Biochemistry of Zooxanthellae Symbiotic with Marine Coelenterates. III. The Effect of Homogenates of Host Tissues on the Excretion of Photosynthetic Products *in Vitro* by Zooxanthellae from Two Marine Coelenterates Proc. R. Soc. Lond. B Biol. Sci 1971 177 251 264

[52] Gates RD Hoegh-Guldberg O McFall-Ngai MJ Bil KY Muscatine L Free Amino Acids Exhibit Anthozoan “Host Factor” Activity: They Induce the Release of Photosynthate from Symbiotic Dinoflagellates *in Vitro* Proc. Natl. Acad. Sci. USA 1995 92 7430 7434 11607567 10.1073/pnas.92.16.7430 PMC41353

[53] Withers KJT Grant AJ Hinde R Effects of Free Amino Acids on the Isolated Symbiotic Algae of the Coral *Plesiastrea versipora* (Lamarck): Absence of a Host Release Factor Response Comp. Biochem. Physiol. Part A Mol. Integr. Physiol 1998 120 599 607

[54] Cook CB Davy SK Are Free Amino Acids Responsible for the ‘Host Factor’ Effects on Symbiotic Zooxanthellae in Extracts of Host Tissue? Hydrobiologia 2001 461 71 78

[55] Grant AJ Remond M Hinde R Low Molecular-Weight Factor from *Plesiastrea versipora* (Scleractinia) That Modifies Release and Glycerol Metabolism of Isolated Symbiotic Algae Mar. Biol 1998 130 553 557

[56] Grant AJ Remond M Withers KJT Hinde R Inhibition of Algal Photosynthesis by a Symbiotic Coral Hydrobiologia 2001 461 63 69

[57] Martin S Parton R Lipid Droplets: A Unified View of a Dynamic Organelle Nat. Rev. Mol. Cell Biol 2006 7 373 16550215 10.1038/nrm1912

[58] Umlauf E Csaszar E Moertelmaier M Schuetz GJ Parton RG Prohaska R Association of Stomatin with Lipid Bodies J. Biol. Chem 2004 279 23699 23709 15024010 10.1074/jbc.M310546200

[59] Imanishi Y Gerke V Palczewski K Retinosomes: New Insights into Intracellular Managing of Hydrophobic Substances in Lipid Bodies J. Cell Biol 2004 166 447 453 15314061 10.1083/jcb.200405110 PMC1360213

[60] Luo YJ Wang LH Chen WNU Peng SE Tzen JTC Hsiao YY Huang HJ Fang LS Chen CS Ratiometric Imaging of Gastrodermal Lipid Bodies in Coral-Dinoflagellate Endosymbiosis Coral Reefs 2009 28 289 301

[61] Tchernov D Gorbunov MY de Vargas C Yadav SN Milligan AJ Haggblom M Falkowski PG Membrane Lipids of Symbiotic Algae Are Diagnostic of Sensitivity to Thermal Bleaching in Corals Proc. Natl. Acad. Sci. USA 2004 101 13531 13535 15340154 10.1073/pnas.0402907101 PMC518791

[62] Wang JT Douglas AE Essential Amino Acid Synthesis and Nitrogen Recycling in an Alga-Invertebrate Symbiosis Mar. Biol 1999 135 219 222

[63] Muscatine L D’Elia CF The Uptake, Retention, and Release of Ammonium by Reef Corals Limnol. Oceanogr 1978 23 725 734

[64] D’Elia CF Domotor SL Webb KL Nutrient Uptake Kinetics of Freshly Isolated Zooxanthellae Mar. Biol 1983 75 157 167

[65] Wang JT Douglas AE Nitrogen Recycling or Nitrogen Conservation in an Alga-Invertebrate Symbiosis? J. Exp. Biol 1998 201 2445 2453 9679106 10.1242/jeb.201.16.2445

[66] Franzisket L Uptake and Accumulation of Nitrate and Nitrite by Reef Corals Naturwissenschaften 1973 60 552

[67] Leggat W Hoegh-Guldberg O Dove S Yellowlees D Analysis of an EST Library from the Dinoflagellate (*Symbiodinium* sp.) Symbiont of Reef-Building Corals J. Phycol 2007 43 1010 1021

[68] Marubini F Davies PS Nitrate Increases Zooxanthellae Population Density and Reduces Skeletogenesis in Corals Mar. Biol 1996 127 319 328

[69] Kuhl M Cohen Y Dalsgaard T Jorgensen BB Revsbech NP Microenvironment and Photosynthesis of Zooxanthellae in Scleractinian Corals Studies with Microsensors for O_2_, pH and Light Mar. Ecol. Prog. Ser 1995 117 159 172

[70] Levy O Achituv Y Yacobi YZ Stambler N Dubinsky Z The Impact of Spectral Composition and Light Periodicity on the Activity of Two Antioxidant Enzymes (SOD and CAT) in the Coral *Favia favus* J. Exp. Mar. Biol. Ecol 2006 328 35 46

[71] Rees TAV Fitt WK Baillie B Yellowlees D A Method for Temporal Measurement of Haemolymph Composition in the Giant Clam Symbiosis and Its Application to Glucose and Glycerol Levels During a Diel Cycle Limnol. Oceanogr 1993 38 213 217

[72] Leggat W Marendy EM Baillie B Whitney SM Ludwig M Badger MR Yellowlees D Dinoflagellate Symbioses: Strategies and Adaptations for the Acquisition and Fixation of Inorganic Carbon Funct. Plant Biol 2002 29 309 322 10.1071/PP01202 32689478

[73] Nakamura H Kobayashi J Hirata Y Separation of Mycosporine-Like Amino-Acids in Marine Organisms Using Reversed-Phase High-Performance Liquid-Chromatography J. Chromatogr 1982 250 113 118

[74] Yakovleva I Hidaka M Diel Fluctuations of Mycosporine-Like Amino Acids in Shallow-Water Scleractinian Corals Mar. Biol 2004 145 863 873

[75] Banaszak AT LaJeunesse TC Trench RK The Synthesis of Mycosporine-Like Amino Acids (MAAs) by Cultured, Symbiotic Dinoflagellates J. Exp. Mar. Biol. Ecol 2000 249 219 233 10841936 10.1016/s0022-0981(00)00192-1

[76] Korbee N Houvinen P Figueroa FL Aguilera J Karsten U Availability of Ammonium Influences Photosynthesis and the Accumulation of Mycosporine-Like Amino Acids in Two *Porphyra* Species (Bangiales, Rhodophyta) Mar. Biol 2005 146 645 654

[77] Kashman Y Groweiss A Carmely S Kinamoni Z Czarkie D Rotem M Recent Research in Marine Natural Products from the Red Sea Pure Appl. Chem 1982 54 1995 2010

[78] Cuif JP Dauphin Y Freiwald A Gautret P Zibrowius H Biochemical Markers of Zooxanthellae Symbiosis in Soluble Matrices of Skeleton of 24 Scleractinia Species Comp. Biochem. Physiol 1999 123 269 278

[79] Boroujerdi Arezue FB Vizcaino Maria I Meyers A Pollock Elizabeth C Huynh Sara L Schock Tracey B Morris Pamela J Bearden Daniel W NMR-Based Microbial Metabolomics and the Temperature-Dependent Coral Pathogen *Vibrio coralliilyticus* Environ. Sci. Technol 2009 43 7658 7664 19921875 10.1021/es901675w

[80] Sumner LW Amberg A Barrett D Beale MH Beger R Daykin CA Fan TWM Fiehn O Goodacre R Griffin JL Proposed Minimum Reporting Standards for Chemical Analysis Metabolomics 2007 3 211 221 10.1007/s11306-007-0082-2 PMC3772505 24039616

[81] Hines A Oladiran GS Bignell JP Stentiford GD Viant MR Direct Sampling of Organisms from the Field and Knowledge of Their Phenotype: Key Recommendations for Environmental Metabolomics Environ. Sci. Technol 2007 41 3375 3381 17539552 10.1021/es062745w

[82] Kluender C Sans-Piche F Riedl J Altenburger R Haertig C Laue G Schmitt-Jansen M A Metabolomics Approach to Assessing Phytotoxic Effects on the Green Alga *Scenedesmus vacuolatus* Metabolomics 2009 5 59 71

[83] Stat M Loh WKW LaJeunesse TC Hoegh-Guldberg O Carter DA Stability of Coral-Endosymbiont Associations During and after a Thermal Stress Event in the Southern Great Barrier Reef Coral Reefs 2009 28 709 713

[84] Rowan R Coral Bleaching: Thermal Adaptation in Reef Coral Symbionts Nature 2004 430 742 15306800 10.1038/430742a

[85] Abrego D Ulstrup KE Willis BL van Oppen MJH Species-Specific Interactions between Algal Endosymbionts and Coral Hosts Define Their Bleaching Response to Heat and Light Stress Proc. R. Soc. Lond. B Biol. Sci 2008 275 2273 2282 10.1098/rspb.2008.0180 PMC2603234 18577506

[86] Cantin N van Oppen M Willis B Mieog J Negri A Juvenile Corals Can Acquire More Carbon from High-Performance Algal Symbionts Coral Reefs 2009 28 405 414

[87] Kim JD Lee CG Systemic Optimization of Microalgae for Bioactive Compound Production Biotechnol. Bioprocess Eng 2005 10 418 424

[88] Kortschak RD Samuel G Saint R Miller DJ Est Analysis of the Cnidarian Acropora Millepora Reveals Extensive Gene Loss and Rapid Sequence Divergence in the Model Invertebrates Curr. Biol 2003 13 2190 2195 14680636 10.1016/j.cub.2003.11.030

[89] Meyer E Davies S Wang S Willis BL Abrego D Juenger TE Matz MV Genetic Variation in Responses to a Settlement Cue and Elevated Temperature in the Reef-Building Coral *Acropora millepora* Mar. Ecol. Prog. Ser 2009 392 81 92

[90] Voolstra CR Sunagawa S Schwarz JA Coffroth MA Yellowlees D Leggat W Medina M Evolutionary Analysis of Orthologous cDNA Sequences from Cultured and Symbiotic Dinoflagellate Symbionts of Reef-Building Corals (Dinophyceae: *Symbiodinium*) Comp. Biochem. Physiol. Part D Genomics Proteomics 2009 4 67 74 10.1016/j.cbd.2008.11.001 20403741

[91] Santos EM Ball JS Williams TD Wu HF Ortega F Van Aerle R Katsiadaki I Falciani F Viant MR Chipman JK Tyler CR Identifying Health Impacts of Exposure to Copper Using Transcriptomics and Metabolomics in a Fish Model Environ. Sci. Technol 2010 44 820 826 20020678 10.1021/es902558k

